# Compensations to auditory feedback perturbations in congenitally blind and sighted speakers: Acoustic and articulatory data

**DOI:** 10.1371/journal.pone.0180300

**Published:** 2017-07-05

**Authors:** Pamela Trudeau-Fisette, Mark Tiede, Lucie Ménard

**Affiliations:** 1 Laboratoire de Phonétique, Université du Québec à Montréal, Center For Research on Brain, Language, and Music, Montreal, Quebec, Canada; 2 Haskins Laboratories, New Haven, Connecticut, United States; Sun Yat-Sen University, CHINA

## Abstract

This study investigated the effects of visual deprivation on the relationship between speech perception and production by examining compensatory responses to real-time perturbations in auditory feedback. Specifically, acoustic and articulatory data were recorded while sighted and congenitally blind French speakers produced several repetitions of the vowel /ø/. At the acoustic level, blind speakers produced larger compensatory responses to altered vowels than their sighted peers. At the articulatory level, blind speakers also produced larger displacements of the upper lip, the tongue tip, and the tongue dorsum in compensatory responses. These findings suggest that blind speakers tolerate less discrepancy between actual and expected auditory feedback than sighted speakers. The study also suggests that sighted speakers have acquired more constrained somatosensory goals through the influence of visual cues perceived in face-to-face conversation, leading them to tolerate less discrepancy between expected and altered articulatory positions compared to blind speakers and thus resulting in smaller observed compensatory responses.

## Introduction

### Speech production and perception in congenitally blind individuals

In the past five decades, researchers have established that speech perception implies the existence of other sources of sensory information, especially auditory and visual sources [1–3]. The McGurk effect [4], a perceptual illusion created by inconsistent auditory and visual information, clearly demonstrates the influence of vision on speech perception. Much less is known, however, about the influence of vision on speech production. In a series of studies that investigated the role of vision on the control of speech, it was previously established that congenitally blind speakers use acoustic and articulatory strategies that are significantly different from those of their sighted peers [5–8]. At the acoustic level, it has been shown that blind individuals produced significantly longer vowels than their sighted peers [6,7,9] and that sighted speakers produced vowels that were spaced further apart in the vowel space compared to congenitally blind speakers [6]. At the articulatory level, these authors demonstrate that blind speakers produced significantly smaller lip rounding than sighted speakers. While investigating the production of focused constituents, Ménard et al. [10] observed that, unlike sighted speakers who mostly use lip movements to increase perceptual saliency, congenitally blind speakers have exploited a different articulatory strategy to ensure intelligibility. Having no access to visual cues, they instead use tongue movements to enhance saliency. Similar results were reported by Trudeau-Fisette et al. [11] and Ménard et al. [7] who showed that sighted speakers primarily used their lips to produce clear speech while blind speakers prioritized the use of their tongue. Thus access to the visual channel affects control of the speech articulators, and consequently the related acoustic targets. For sighted speakers, lip positions are heavily weighted in the speech goal, since they are tightly linked to audiovisual saliency. In the hyperarticulation associated with efforts to increase perceptual saliency, this emerges in increased movement of the lips, for sighted speakers, and increased movements of the lips and the tongue for blind speakers.

However, in conditions where speech intelligibility is jeopardized, different patterns are observed. In a study where congenitally blind and sighted French speakers were required to produce speech in a fast condition [12], we showed that blind speakers produce a larger number of vowels for which spectral components are within the acoustic areas that correspond to the canonical phonemic target. These results suggested that in order to maintain saliency, blind speakers focused on safeguarding their auditory distinctiveness, whereas sighted speakers, who know that intelligibility can be transmitted through visual cues, accorded less weight to auditory targets. In other words, when intelligibility is compromised, acoustic goals are more substantially preserved for blind speakers than for sighted speakers.

In another study that compared sighted and blind speakers, we used the paradigm of lip perturbation to investigate the extent to which visual deprivation affects articulatory compensations [13]. Ultrasound imaging was used to measure tongue shape and position when producing the vowel /u/ before, during, and after the insertion of a lip tube. Since the lip tube prevents the lips from rounding, this condition is well-suited to observe the ability of speakers to develop an appropriate strategy to compensate for the lip opening induced by the tube (e.g., place the tongue in the back of the mouth in a lower position). The study showed that during the compensatory response, blind speakers moved their tongue significantly more than their sighted peers. This larger articulatory compensation in blind participants suggests that they tolerated larger discrepancies between the expected somatosensory consequence and the actual produced tongue position, while they tolerated less auditory error. However, in this experiment, only tongue movement was studied, in response to the lip-tube perturbation.

### The role of multimodal sensory feedback in speech production

According to Perkell [14], Tourville and Guenther [15] and Guenther and Vladusich [16], speech goals are specified in multimodal sensory dimensions, the most important ones being the auditory and somatosensory dimensions. Articulatory gestures are controlled through feedforward commands and feedback mechanisms. During speech development, auditory and somatosensory feedback is used to gradually calibrate feedforward commands that specify the relationships between articulatory positions and acoustic or somatosensory output (for more details on internal models and on the debate concerning their exact nature, see [17–21]). In adults, speech motor control primarily relies on feedforward commands that predict the sensory consequences of articulatory commands [22–25]. These expected sensory consequences are compared with actual feedback, generating errors that lead to adjustment of articulatory commands [26–29]. These studies have emphasized the complexity of the interaction between auditory and somatosensory feedback in adult speech production.

In normal-hearing speakers, the study of sensorimotor adaptation, defined as an individual’s ability to gradually modify his/her motor commands to compensate for changes in the environment, has been fruitful for evaluating the importance of auditory feedback in speech production. Experiments with perturbations in which auditory feedback is unexpectedly modified have shown that speakers produce changes in subsequent speech production to compensate for the altered auditory feedback [30–37]. The exact mechanisms involved in compensatory behavior is a result of multiple processes in speech production, at least including setting sensory goals, converting to concrete articulatory plans, predicting sensory consequences by forward models, perception of feedback, comparison between prediction and feedback, and inverse modeling for motor command updating.

Many studies that looked more specifically at the manipulation of formant frequencies [30,34,38,39], have demonstrated that when speakers hear feedback in which a formant is shifted in real-time, they adapt their production by changing the formant in the opposite direction to the perturbation [33,34,38,40–44]. Several studies have shown that the level of compensation seems to be linked to the level of the formant alteration, even when speakers are asked not to compensate [39,45]. However, participants only partially adapt their speech production in response to the auditory perturbation, even when it would be physiologically possible for them to compensate completely [33,39,45–48]. Beyond a certain degree of acoustic shift, participants often reach a plateau of compensation, which can also lead to a decrease in compensation.

Moreover, compensation is not immediate [45,47,48]. For instance, when shifting F1 on isolated vowels, Purcell and Munhall [39] observed that the compensation only appeared when a certain level of alteration was reached. They proposed that for an alteration of 200 Hz, the threshold is, on average, 76 Hz, although they reported large between-subject variability. This suggests that our production-perception model accepts some variability, beyond which an adjustment of the production mechanisms would operate.

The between-speaker variability in compensatory responses to auditory perturbation described earlier can be explained by many factors, including auditory perceptual acuity. Villacorta [49] and Villacorta et al. [34] showed that there was a significant correlation between perceptual acuity and the degree of compensation to a formant shift. Indeed, subjects with better auditory skills compensated their speech production to a greater extent in response to a manipulation of F1. Since, in this framework, better auditory skills are linked to enhanced speech production contrasts [50], this result indirectly suggests that better compensatory responses are associated with better production abilities. However, studies of compensation to auditory perturbation conducted with speakers with speech deficits have led to a contradictory interpretation. Indeed, in a study of compensation to pitch-shift, adults with Parkinson's disease produced larger compensatory responses compared to control participants. This result was interpreted as indicating a lack of control or an inability to bring the response back to the intended target in individuals with Parkinson's disease [37]. However, the participants used an altered speech production and perception mechanism and results might reflect different mechanisms involved in compensation. In speakers with Parkinson's disease, speech is often perceived as being less intelligible that it is for control participants. Thus, the speech goal that is perturbed and compensated for is different in both speaker groups. Nevertheless, these contrasting patterns of results for normal and abnormal speech speak to the complexity of the mechanisms involved in the auditory perturbation paradigm.

Along the same lines, it appears that the threshold at which a compensatory behavior appears varies depending on the phonological organization of the language [36] and on the location of the perceived altered sound with respect to its category boundary [36,51] For example, if increasing F1 in /e/ by 200 Hz pushes the token outside the acoustic region associated with this vowel so that it sounds like /ɛ/, the speaker might be forced to compensate more than with smaller F1 shifts that maintain the heard vowel within the /e/ category.

Variations in compensatory responses to acoustic feedback perturbations can also be ascribed to the competing demands of somatosensory feedback [33,43,51,52]. Indeed, compensation in the auditory domain requires modifications of the articulatory strategies. The compensation in the auditory domain will then cause a discrepancy in the somatosensory/articulatory domain. Therefore, the partial auditory compensation following the auditory perturbation is the result of balance between auditory compensation and tolerance of discrepancy in the somatosensory domain. In order to produce greater auditory compensatory responses, the speaker would have to tolerate a larger discrepancy in the somatosensory domain.

Another set of studies looked at participants who were required to produce speech under conditions of compatible or incompatible auditory and jaw perturbations [52,53] In Feng et al. [53], when compatible auditory and somatosensory perturbations were applied simultaneously (F1 shifted up and the jaw pushed downwards), the subjects compensated for both perturbations, but when incompatible perturbations were applied simultaneously (F1 shifted up and the jaw pushed upwards) the subjects showed significant compensation only for the auditory perturbation. In Lametti et al. [52], a large inter-subject variability was observed: some participants compensated strongly for the auditory perturbation, and little for the jaw perturbation, while the other participants compensated strongly for the jaw perturbation and little for the auditory perturbation. This was true when each perturbation was applied separately as well as when both perturbations were applied simultaneously. Lametti et al. [52] concluded that there is a subject-dependent sensory preference in the specification of the motor goals in speech production, in line with a study by Katseff et al. [51].

Taken together, these studies conducted on adult speakers confirm that goals in speech production are specified in both auditory and somatosensory domains. However, while Feng et al.’s [53] findings provide support for the hypothesis of a general dominance of the auditory specification over the somatosensory one, Lametti et al.’s [52] observations tend at a first glance to be incongruent with this view and instead suggest a subject-specific hierarchy between these two types of goals. However, in Lametti et al.’s [52] experiment, the jaw perturbation did not influence the acoustic results, in contrast to what happened in one of the conditions of Feng et al.’s [53] experiment. Lametti et al.’s [52] experiment did not show that somatosensory goals are preserved when this preservation endangers auditory goals. Hence, both experiments are compatible with the hypothesis that the acoustic specification of speech goals is hierarchically above the somatosensory component of speech. Lametti et al’s [52] results suggest that there is, in both domains, a limited tolerance for a discrepancy between the specification of the speech goals and the characteristics of the corresponding auditory feedback, and that the relative weights of the tolerance in each domain are subject-specific.

Finally, several authors have noted that compensatory behavior persists even when the acoustic manipulation is completely removed. This long-term adaptation seems to be unrelated to the direction of the manipulation or to the duration of exposure to the altered auditory feedback [34,39,48].

At the articulatory level, compensations in response to a change in the acoustic auditory feedback have been less explored [48,54]. One exception is the study of Feng et al. [53], who asked participants to produce the /ɛ/ vs. /æ/ contrast while F1 was shifted up or down, simultaneously combined with an upward or downward force applied on the jaw. They measured tongue, jaw, and lip positions using electromagnetic articulography (EMA) and found that when F1 was shifted up, participants significantly raised their tongue tip and moved their jaw to lower their produced F1. Taken together, these studies suggest that speakers actively compensate for altered auditory feedback by changing their tongue and jaw articulatory positions (which generates discrepancies between expected and actual somatosensory feedback), to produce acoustic values close to the unperturbed target (which also generates some discrepancies between expected and actual auditory feedback since compensation is never complete).

In the current study, we used a paradigm of auditory perturbation to investigate the relationship between visual deprivation and auditory feedback in congenitally blind speakers. Acoustic and articulatory data were collected during the production of /ø/ “eu” as in “feu” (*fire*) for which speakers heard altered F2 values. First, we hypothesized that at the acoustic level, speakers would alter F2 in the opposite direction of the change they perceived, with a larger alteration for blind speakers than for sighted speakers (cf. [13]). Second, we hypothesized that, at the articulatory level, blind speakers would produce larger compensatory changes in tongue positions than sighted speakers. Third, we hypothesized that lip positions would be altered to a lesser extent in blind speakers than in sighted speakers.

## Materials and methods

The study consisted of two experiments, a speech perception task and a speech production task, which were conducted in a single session. This research was approved by the Université du Québec à Montréal's Institutional Review Board (no 2012-05-4.3), and all participants gave written, informed consent.

### Participants

Ten congenitally blind speakers (4 females, 6 males; mean age 41.7 years) and ten age-matched sighted speakers (5 females, 5 males; mean age 43.2 years) were tested in a soundproof room at the Phonetics Laboratory of the Université du Québec à Montréal. All subjects had French as their first language and reported having no speech or language impairments. Pure-tone detection thresholds were assessed using an adaptive method with supra-auricular earphones. All participants had detection thresholds below 25 dB HL at every frequency tested (250 Hz, 500 Hz, 1000 Hz, 2000 Hz, 4000 Hz, and 8000 Hz), which corresponds to normal hearing. Characteristics of the blind participants are shown in Table 1.

**Table 1:** Characteristics of the 10 blind speakers.

Subject	Gender	Age	Etiology of blindness	Vision at birth	Current vision
S1B	F	48	retinitis pigmentosa	U	R.E. = 3/210 L.E. = 0
S2B	F	40	congenital cataract	U	R.E. = 0 L.E. = 6/1260
S3B	F	26	U	U	U (total blindness)
S4B	M	52	optic atrophy	total blindness	R.E. = 0 L.E. = 0
S5B	M	40	detachment of the retina	U	R.E. = 2/180 L.E. = 2/105
S6B	M	42	congenital cataract and congenital glaucoma	U	U (total blindness)
S7B	F	51	retinitis pigmentosa	total blindness	R.E. = 2/400 L.E. = 2/400
S8B	F	45	congenital cataract	total blindness	U (total blindness)
S9B	M	42	congenital glaucoma	U	R.E. = 2/180 L.E. = 3/180
S10B	F	45	congenital cataract	total blindness	R.E. = 0 L.E. = 0

**L.E.** = left eye; **R. E.** = right eye; **U** = undetermined.

### Perception experiment

During the perception task, participants had to categorize, through in-ear headphones (Etymotic Mc5), 10 repetitions of each of the 12 members of a synthesized /e–ø/ continuum, synthesized in equal steps with the Maeda model [55]. This continuum contained 12 unique tokens for which the first four formants corresponded to those between the natural endpoint tokens of /e/ and /ø/. Formant and bandwidth values are shown in Table 2. Thus, participants had to categorize 120 tokens as a forced choice between the proposed vowel options /e/ and / ø/. Once they selected a vowel, they had to rate the quality on a discrete scale labelled from 1 (poor) to 7 (excellent). During the experiment, each token was presented randomly. The perception experiment was performed using Praat (version 5.3.80). To ensure that the same experimental protocol was used for the two groups, no participant was permitted to see the computer screen during the task. To indicate their choices, they needed to tell the experimenter so that she could select the mentioned vowel and quality level. Both blind and sighted speakers received the same instructions orally by the same experimenter. Sighted speakers did not have any visual cues that would have provided further information concerning the tasks. Probit modelling was used to obtain psychometric labelling functions for each participant, from which the 50% crossover boundary and labelling slope were identified. ANOVAs were conducted on boundaries and slopes with speaker group as the independent variables.

**Table 2:** Formant and bandwith values of the synthesized stimuli used in the perceptual task.

	Formants					Bandwiths				
	F1	F2	F3	F4	F5	B1	B2	B3	B4	B5
stim1	364	1922	2509	3550	4000	48	55	60	50	100
stim2	364	1892	2469	3500	4000	48	55	60	50	100
stim3	364	1862	2429	3450	4000	48	55	60	50	100
stim4	364	1832	2389	3400	4000	48	55	60	50	100
stim5	364	1802	2349	3350	4000	48	55	60	50	100
stim6	364	1772	2309	3300	4000	48	55	60	50	100
stim7	364	1742	2269	3250	4000	48	55	60	50	100
stim8	364	1712	2229	3200	4000	48	55	60	50	100
stim9	364	1682	2189	3150	4000	48	55	60	50	100
stim10	364	1652	2149	3100	4000	48	55	60	50	100
stim11	364	1622	2109	3050	4000	48	55	60	50	100
stim12	364	1592	2069	3000	4000	48	55	60	50	100

### Production experiment

#### Recording procedures

Acoustic and articulatory recordings of several instances of the vowel /ø/ “eux” (*them*) were collected simultaneously. Sound signal was recorded via a high-quality Audio-Technica microphone (Omnidirectional condenser Headworn microphone, model number BP892) and digitized at 44100 Hz using a Delta 1010 LT sound card. Articulatory measurements were captured through an electromagnetic articulography system (AG500, Carstens Medizinelektronik, Carstens, 2006). This device allows, via small sensors, the three-dimensional motion tracking of visible and non-visible articulators (such as the lips, jaw, and tongue). Although some limitations of this method have been identified regarding the computing of sensor localization in certain areas of the measurement field [56], it has been demonstrated that this method, which allows a high temporal resolution tracking of internal and external articulators, remains a reliable one for speech articulatory measurements when it is used accurately and when data are carefully processed in consideration of the spatial error distribution [57].

Prior to the main experiment, readings from a reference static pose were recorded while the participant remained immobile for a few seconds. This identified the neutral position of reference sensors attached to the gumline at the upper incisors, and left and right mastoid processes used for characterizing head position. During post-processing this reference position was used to correct for any head movement during data collection by rotating each sensor trajectory to the original reference and translating to a coordinate system centered on the upper incisors, thus allowing comparison of sensor displacement across subjects.

**Fig 1:**
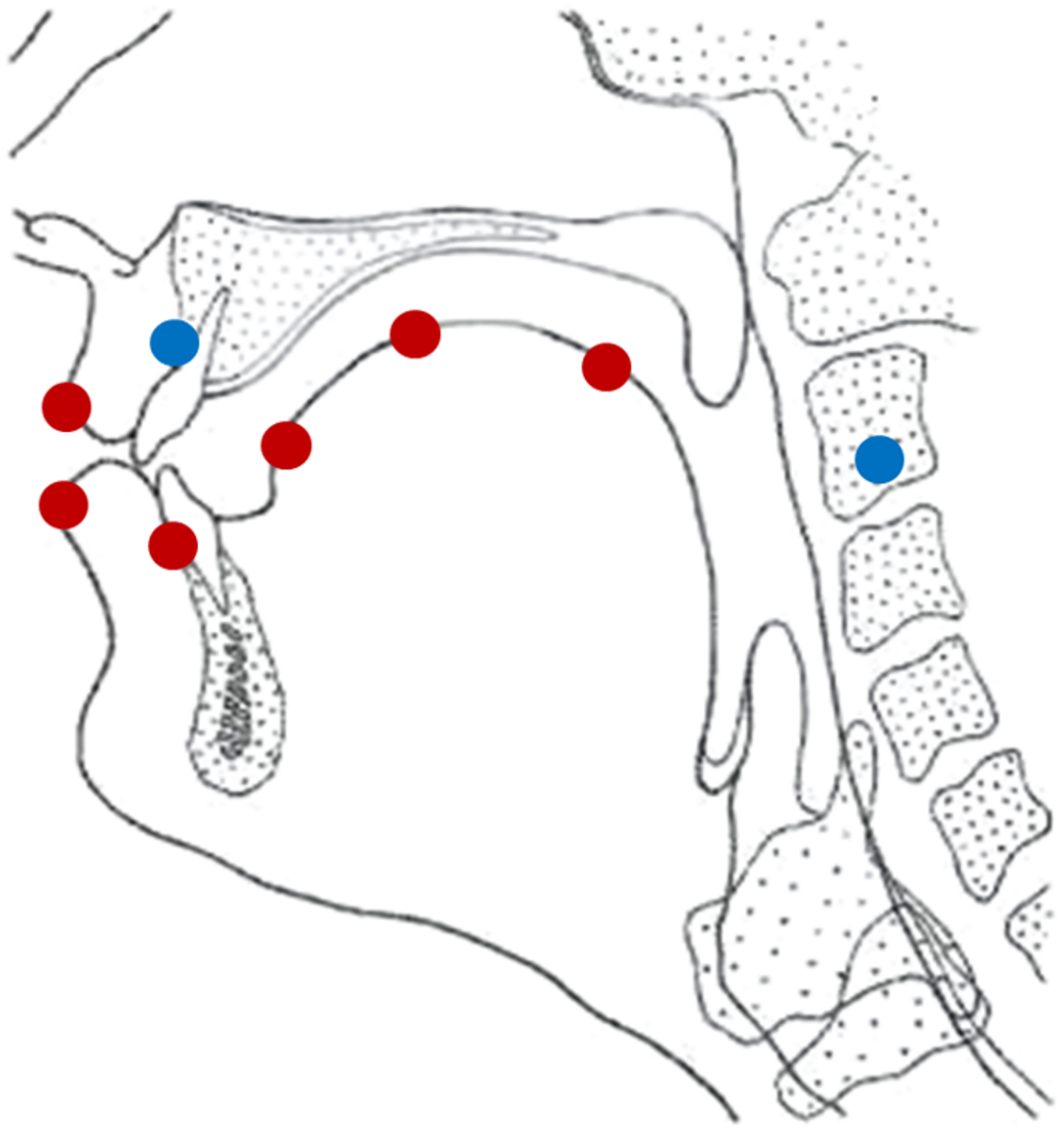
Sensor locations during the experiment. Red ones were used for the analysis of articulators of speech, while blue ones served as reference points.

A total of nine sensors were used to collect the articulatory data. Fig 1 shows the location of each of the sensors. Six sensors (in red in Fig 1) were placed midsagittaly to track the speech articulators: three on the tongue, one on the jaw (gumline at lower incisors), and one on the upper and lower lips. The other three served as references (in blue in Fig 1) for head correction. The sensors were attached with dental adhesive (Cyano Veener).

#### Formant shifting

Equipment The formant-shifting equipment for the experiment was similar to that used by Munhall et al. [45] MacDonald et al. [47], and Mitsuya et al. [36]. A speech signal was recorded through a microphone (Audio-technica BP892). In order to improve the spectral resolution and to facilitate the formant tracking, the microphone signal was amplified (Tucker-David Technologies MA 3 microphone amplifier) and low-pass filtered with a cut-off frequency of 4.5 kHz (Frequency Devices 901 filter). It was then digitized at 10 kHz, and filtered in real-time to produce formant shifts (National Instruments PXIe-1071embedded controller). In order to limit the perception of natural acoustic feedback through mouth to ear airborne signal, the altered signal (80 dBA SPL) was combined with a pink noise (50 dBA SPL) and returned to the speaker through in-ear headphones (Etymotic Mc5).

Estimating model order To estimate the model order, a parameter that defines the number of coefficients used in the LPC auto-regressive analysis, we collected 42 utterances of the targeted word “eux” (*them*), corresponding to the vowel /ø/. Both sighted and blind speakers were asked to speak as normally as possible and were instructed to wait for a physical cue (light touch on their shoulder) to produce the prompted word. Those utterances were then analyzed with different model orders, ranging from 8 to 12. For each individual, the best model was selected based on lowest variance in the second formant frequency, over a 25 ms segment around the vowel mid-point [47]. This protocol insures that the best formant tracking parameters are used for each participant. With this customized detection of the formant values, the acoustic manipulation is carried out optimally for every individual.

Voicing detection and online formant shift As described in Munhall et al. [45], MacDonald et al. [47] and Mitsuya et al. [36], the voicing detection was done using a statistical, amplified-threshold technique and formant shifting was performed in real-time using an infinite impulse response filter. Prior to the experiment-data collection, an online estimation of formant frequencies was obtained via an iterative Burg algorithm [58]. Filter coefficients were then computed related to these estimates. To create the shift, a pair of spectral zeroes was placed on the existing formant and a pair of spectral poles was positioned at the desired location of the new formant.

**Fig 2:**
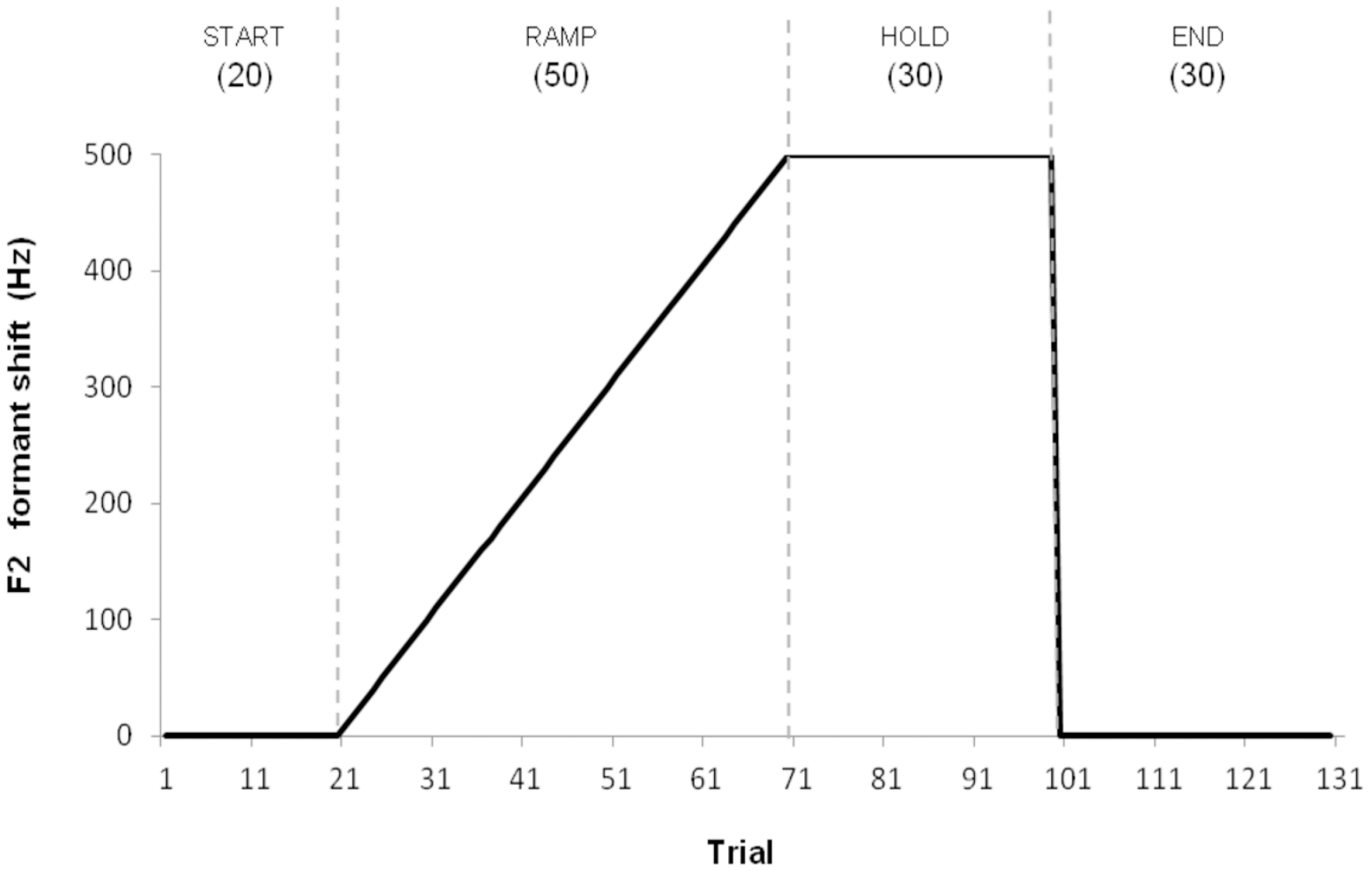
Schematic representation of the formant shift during the experimental phases. Dashed lines indicate the frontiers of the four phases (Start, Ramp, Hold, End).

Experimental phases In the experiment task, the participants had to produce 130 utterances of the rounded vowel “eu” /ø/. To make sure that they were producing the vowel in the appropriate timeframe, participants were again asked to wait for a physical cue (light touch on their shoulder) to produce it. The session was divided into four phases (Fig 2). In the first phase (baseline phase: trials 1–20), participants received normal auditory feedback. In the second phase (ramp-up phase: trials 21–70), participants received altered auditory feedback during which F2 was incrementally increased by 10 Hz over the course of every trial (the other formants were not affected). Therefore, on the 70^th^ trial, participants received an auditory feedback for which F2 was raised by 500 Hz. In the third phase (hold phase: trials 71–100), that 500 Hz shift was simply maintained. At the 101^st^ trial, the perturbation was abruptly removed. Thus, during the last phase (end phase: trials 101–130) participants received normal auditory feedback. In order to maintain the same protocol during the entire experiment, the two phases where the auditory feedback was not manipulated were also amplified and mixed with masking noise.

Offline formant analysis For every target vowel production, a manual segmentation of the vowel was made using Praat (version 5.3.80). Following the segmentation of the 130 utterances of the target vowel, the first four formants were extracted at vowel mid-point using a custom script. We only collected the formant values at the center of each vowel production. If any estimates were incorrectly labeled (e.g., F1 mislabelled as F2, etc.), they were corrected manually.

### Data analysis

For each target vowel, acoustic and articulatory measures were extracted. First, acoustic signals were down-sampled to 22050 Hz, after low-pass filtering (cut-off frequency of 10000 Hz). The first three formant frequencies were then estimated for each vowel, using the linear predictive coding (LPC) algorithm implemented in the Praat speech analysis program. The number of poles ranged from 12 to 18. A 14-ms Hamming window centered at the vowel mid-point was used, with a pre-emphasis factor of 0.98 (pre-emphasis from 50 Hz for a sampling frequency of 22050 Hz). For each speaker, the baseline averages of F1, F2, and F3 values were calculated based on the last 15 utterances of the start phase (utterances 6–20). Utterances 1 to 5 were left out of the baseline average to counter the effect of familiarization of the task (following [46]). Then, each spectral measure (F1, F2, and F3) was represented as a proportion of the speaker’s baseline average. To assess the global impact of auditory perturbation, and to investigate whether it was different depending on the speaker group (blind versus sighted speakers), separate repeated-measure ANOVAs were conducted with F1 ratios, F2 ratios, and F3 ratios as the dependent variables, speaker group (blind or sighted) as the between-subject factor, and experimental phase (start, ramp-up, hold, and end) as a within-subject factor. In this analysis, data were averaged over all trials for each condition. Significant interactions were further explored with post-hoc analyses, and confidence intervals were adjusted for multiple comparisons with the Fisher Least Significant Difference (LSD) test correction.

At the articulatory level, sensor positions were extracted at the same vowel midpoint used for formant estimation. Articulatory measures included x (anterior/posterior) and z (inferior/superior) positions of the upper lip, lower lip, jaw, tongue tip, tongue blade, and tongue body. For each speaker, the baseline averages of x and z values of each sensor were calculated based on the last 15 utterances of the start phase (utterances 6–20). Then, for each speaker, sensor, and trial, the Euclidean distance between the (x, z) coordinates and the average coordinates in the baseline was calculated. This measure provided two-dimensional contrast distances for each sensor during the various experimental phases. Repeated measures of analyses of variance (ANOVA) were conducted with the subject group, the experimental phase, and the sensor as the independent variables. Violations of sphericity were checked using the Greenhouse-Geisser epsilon variable. Since no violation was found, the original degrees of freedom were reported. Effect sizes, corresponding to eta-squared values, and values of Wilk’s lambda are reported.

## Results

### Perception experiment

**Fig 3:**
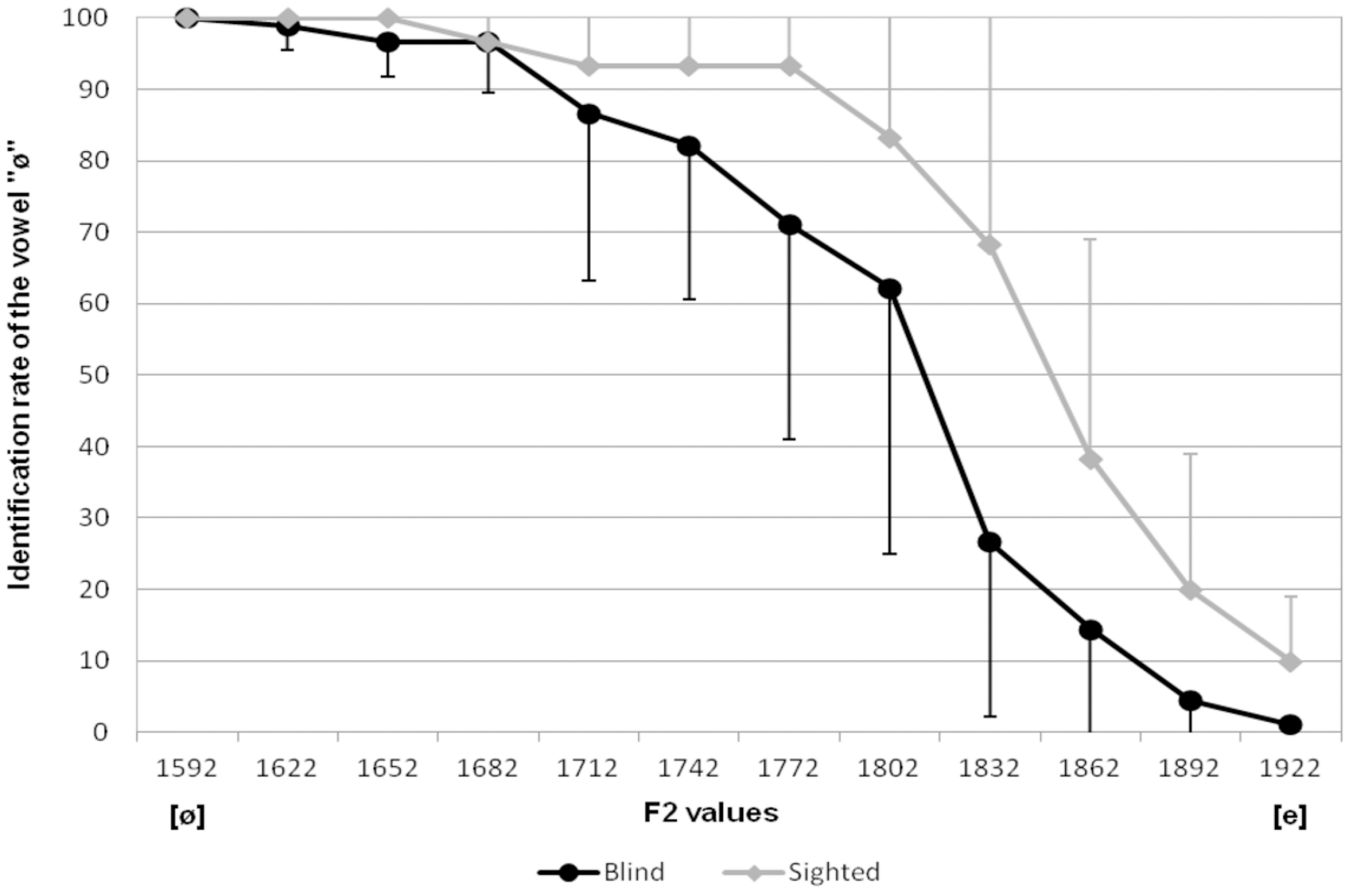
Percent identification of the vowel [ø] for stimuli of the [e-ø] continuum according to its F2 value, for both speaker groups. Error bars indicate standard deviation.

To identify the perceptual abilities of our participants, for each subject, psychometric labelling functions along the /e/-/ø/ continuum were compared. Fig 3 shows average identification scores for both groups. It should be noted that among all participants, 4 sighted speakers and 1 blind speaker had to be excluded since their identification rate did not cross the 50% threshold, which is required to create a valid psychometric curve. Fifty-percent category boundaries and labelling slopes were obtained through Probit modelling, for each speaker. Two one-way ANOVAs were conducted on the 50% boundaries and on the slopes with speaker group as the independent factor. For both variable, no significant effect of speaker group were found ((50% boundaries: F(1,13) = 4.295, p = 0.059) and (slope: F(1,13) = 0.036, p>0.05)).

### Production experiment

#### Acoustic results

**Fig 4:**
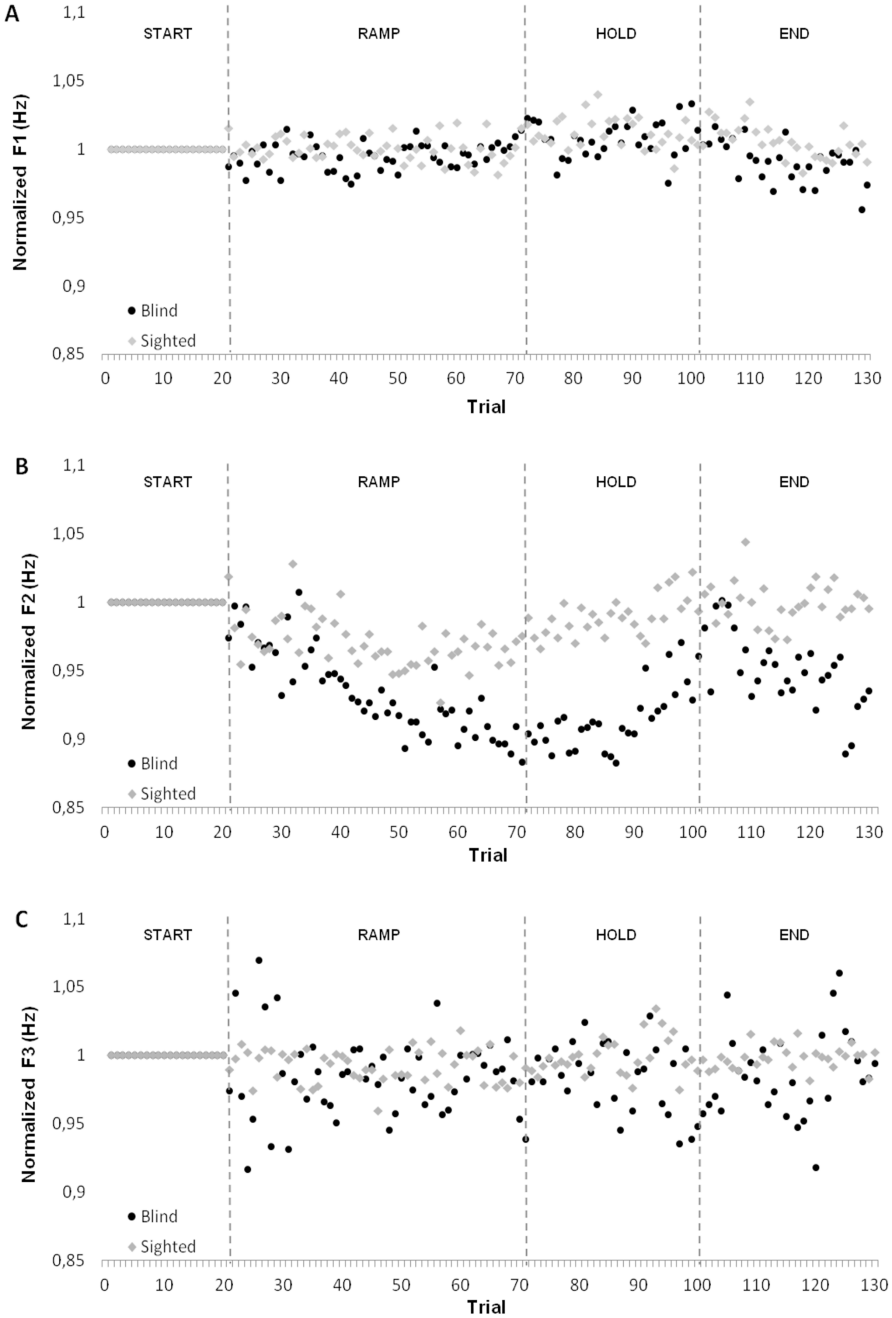
Normalized formant values (A- F1, B- F2, C- F3) average across speaker group. Black dot refers to blind speakers and gray diamond refers to sighted speakers.

Fig 4 displays the normalized F1 (by proportion of speaker’s baseline) (upper panel), F2 (center panel), and F3 (lower panel) values for each utterance and for both speaker groups, averaged across speakers. Since the degree of adaptation is presented in terms of ratio, a value of 1 refers to productions for which there is no compensation. Values greater than 1 indicate that there has been an increase in the observed formant, while values less than 1 denote a decrease of this formant.

To evaluate the acoustic adaptation across experimental phases, the mean value of the baseline phase (utterances 6–20), hold phase (utterances 71–100) and the end phase (utterances 101–130) were calculated (the ramp phase will be discussed later). For each formant, a repeated measures ANOVA was conducted with experimental phase as a within-subject factor and speaker group as a between-subject factor. For F1, neither the experimental phase (F(2, 36) = 0.841, *p*> 0.05) nor the speaker group (F(1,18) = 0.311, *p*> 0.05) were significant. For F2, both the variables had significant effects on F2 values (experimental phase: F(2,36) = 7.205, *p* <0.01; speaker group: F(1,18) = 4.777, *p* <0.05). For the main effect of the experimental phase, a post-hoc analysis with Bonferroni adjustment showed that all participants significantly offset their productions during the holding phase compared to the baseline phase (F(1,18) = 11.153, *p* <0.05) and compared to the end phase (F(1,18) = 8.133, *p* <0.05). Moreover, a significant interaction between the two factors was detected (F(2,36) = 7.323, *p* <0.05). A post-hoc analysis with Bonferroni adjustment revealed that blind participants altered their production significantly more than their sighted peers during the holding phase, compared to the baseline phase (F(1,18) = 6.627, *p* <0.05).

Since the largest compensations in F2 were not observed in the same experimental phase for both speaker groups, we quantified the maximum compensation for each speaker group, regardless of the experimental phases. We calculated the mean value of the 26 productions for which compensations were the most important (which refers to 20% of all productions). A student's T-test revealed a significant difference between the average maximum compensation of the two speaker groups (t(18) = -2.295, *p* <0.05), indicating that, to compensate for the applied acoustic manipulation, blind speakers adapted their productions significantly more than sighted speakers.

We also sought to identify the moment when the participants began to produce utterances for which the F2 value was significantly different from those of the baseline phase. To proceed, we identified the standard error of the baseline phase (utterances 6–20) for each participant. A significant change was declared when a subject produced three consecutive repetitions for which F2 values differed by more than 3 standard errors from those of the baseline phase. For each subject, the level of acoustic manipulation associated with the change point was extracted, and this revealed that sighted speakers started to significantly adapt their vowel production in response to a manipulation of 159 Hz, while the blind speakers significantly adapted their vowel productions after a manipulation of 149 Hz. A t-test for which the magnitude of the manipulation associated with the change point of each participant was considered showed that this difference was not significant (t(18) = -0.159, *p*> 0.05).

For F3, neither the experimental phase effect (F(1.414,36) = 0.231, *p*>0.05) nor the group effect (F(1,18) = 0.177, *p*>0.05) were significant.

#### Articulatory results

**Fig 5:**
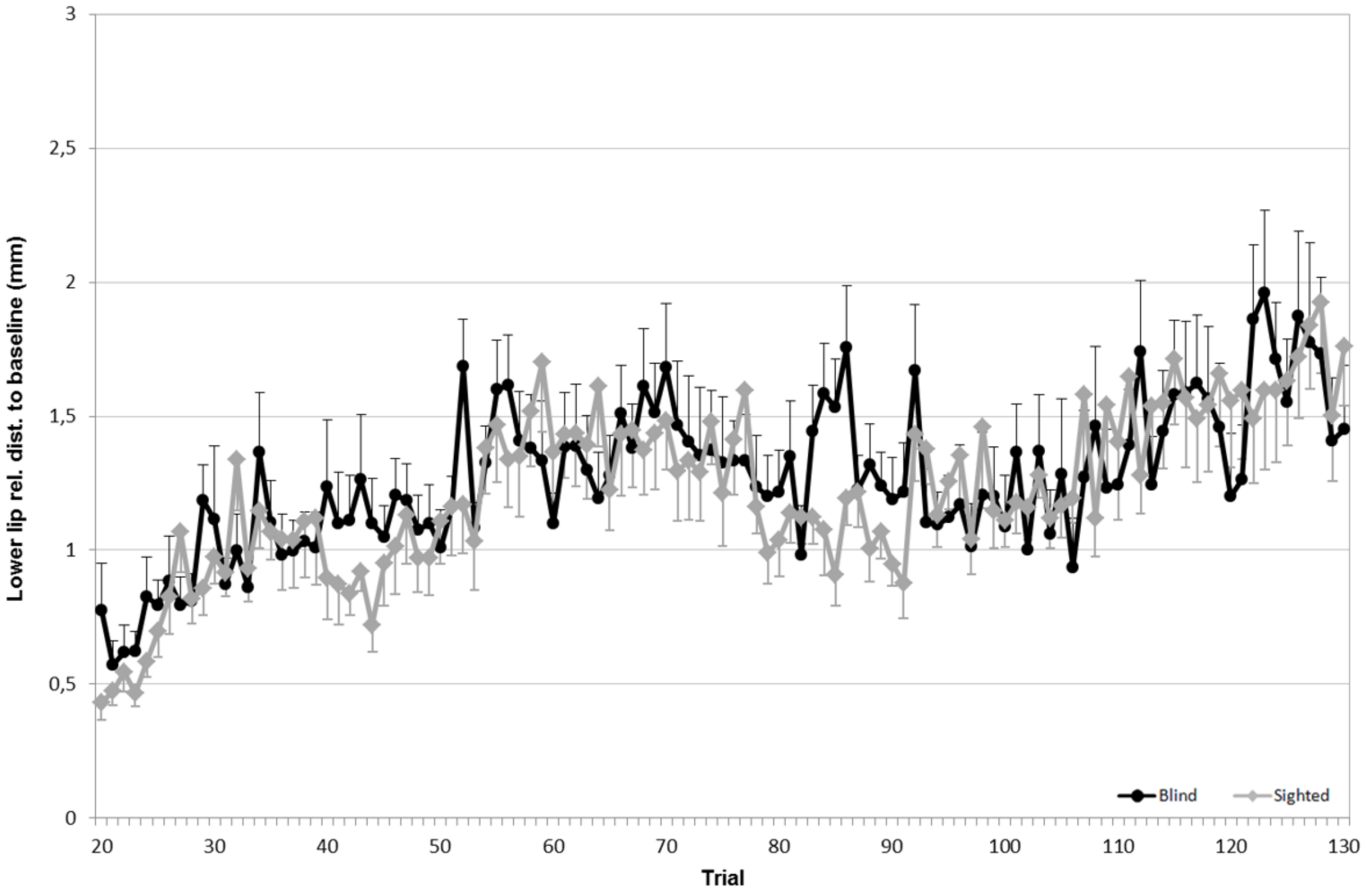
Average Euclidean distance (in mm) relative to the baseline for the lower lip. **Black lines refer to blind speakers while gray lines refer to sighted speakers**. Error bars indicate standard errors.

**Fig 6:**
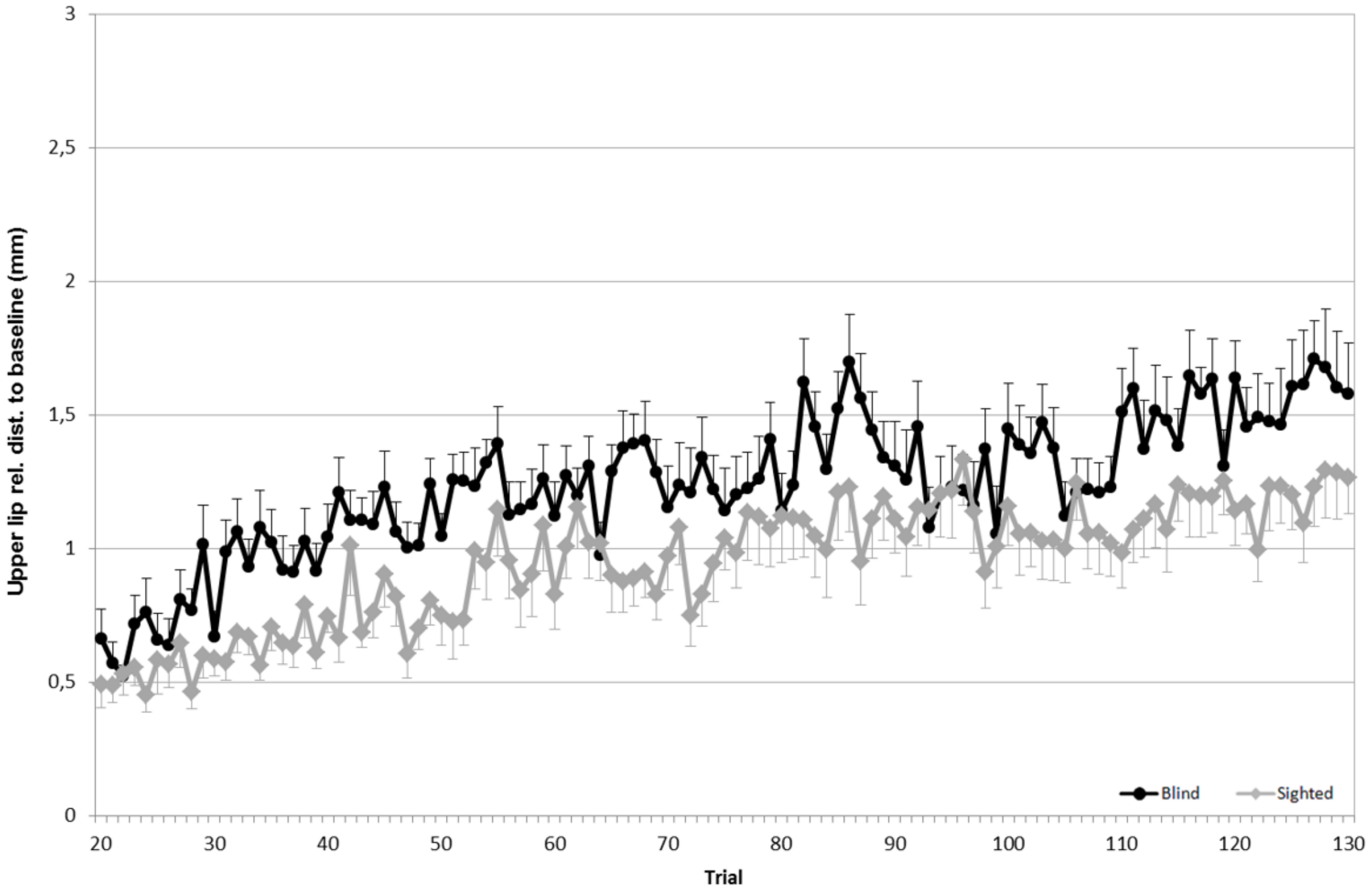
Average Euclidean distance (in mm) relative to the baseline for the upper lip. **Black lines refer to blind speakers while gray lines refer to sighted speakers**. Error bars indicate standard errors.

**Fig 7:**
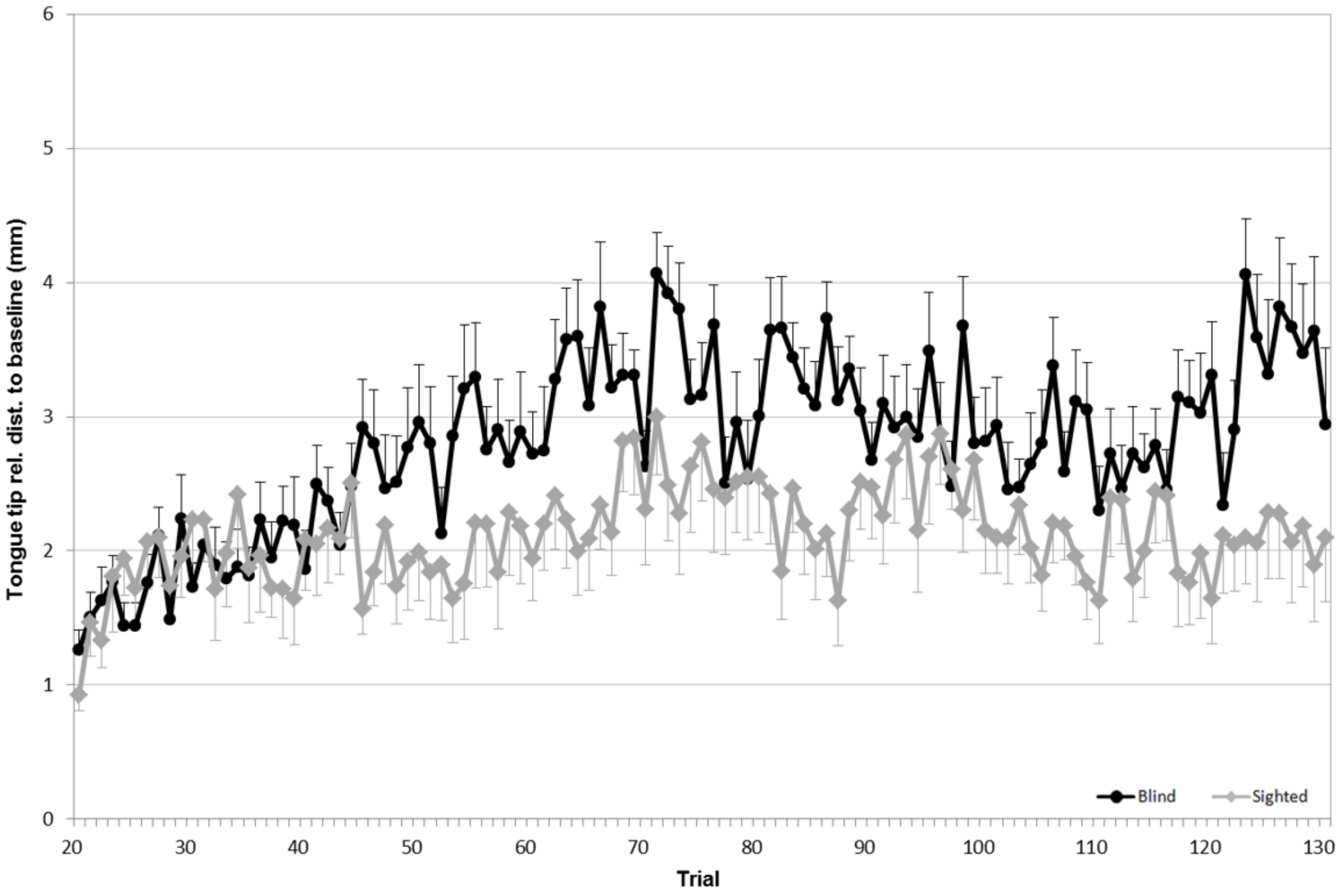
Average Euclidean distance (in mm) relative to the baseline for the tongue tip. **Black lines refer to blind speakers while gray lines refer to sighted speakers**. Error bars indicate standard errors.

**Fig 8:**
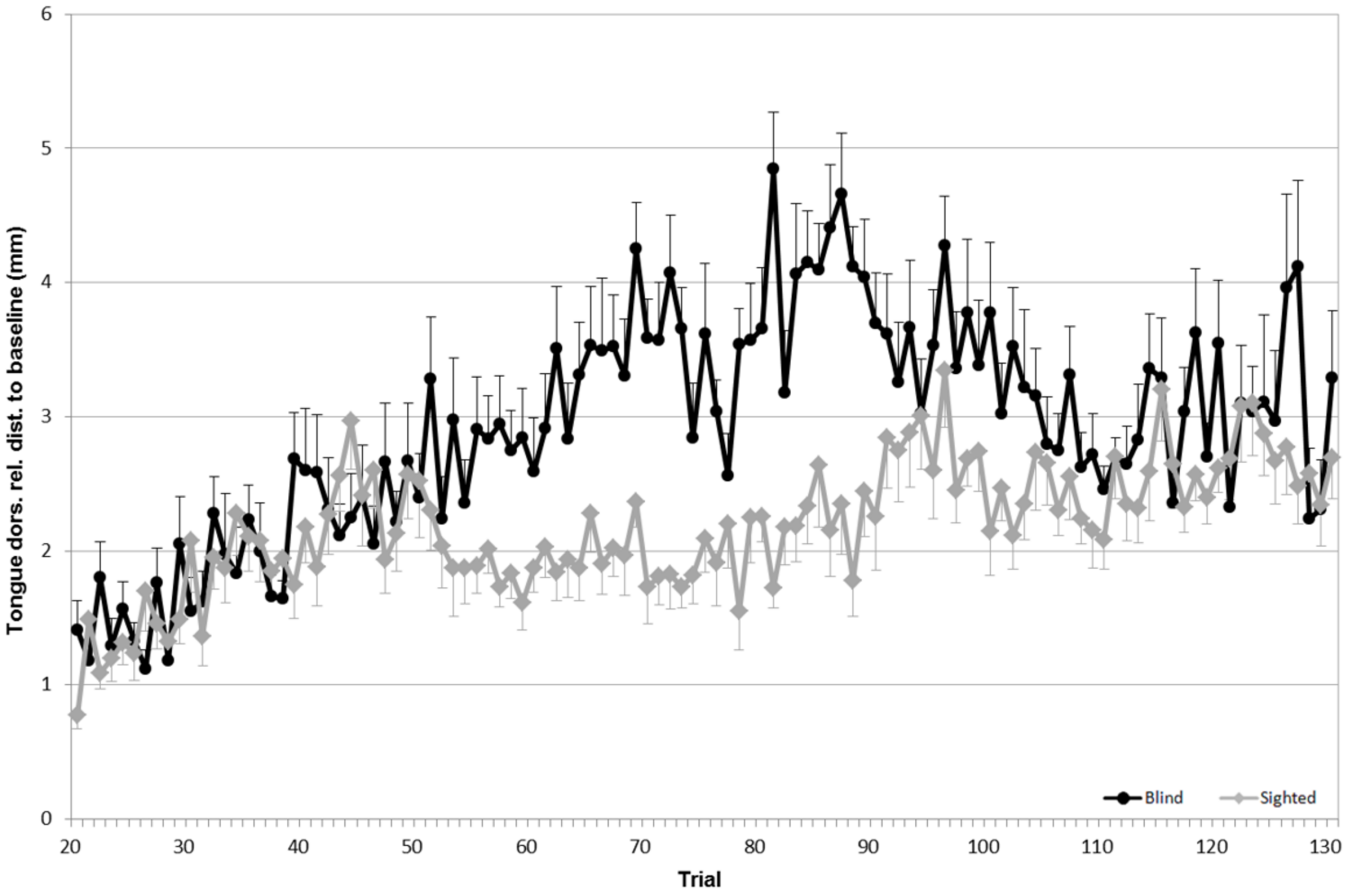
Average Euclidean distance (in mm) relative to the baseline for the tongue dorsum. **Black lines refer to blind speakers while gray lines refer to sighted speakers**. Error bars indicate standard errors.

To evaluate the articulatory strategies used to overcome a given acoustic manipulation, we looked at four articulators: upper lip (UL), lower lip (LL), tongue tip (TT) and tongue dorsum (TD). For both speaker groups, the average Euclidean distances for each sensor (in the x, z dimensions), compared to the baseline phase, are shown in Figs 5 to 8.

Since the degree of articulatory displacements is presented in terms of difference, a value of 0 refers to productions for which no articulatory movement was observed. Values greater than 0 indicate a forward, upward, downward, or backward movement relative to baseline. In regards to the upper lip, as seen in Fig 6, a significant effect of the experimental phase is found (F(3,57) = 15.83; p<0.001). Both speaker groups significantly moved this articulator while going from the baseline phase to the hold phase (F(1,19) = 18.12, *p*<0.01). No statistical difference was observed between the hold phase and the end phase or between the baseline phase and the end phase. A significant effect of the interaction between speaker group and experimental phase was found (F(3,57) = 9.97, p>0.05), with blind speakers moving their upper lip to a greater extent than sighted speakers in the hold phase. Concerning the displacements of the lower lip, no main effect of experimental phase or group was found.

As for the tongue tip, a significant effect of phase was found: both speaker groups moved their tongues when going from the baseline phase to the hold phase (F(3,57) = 12.05, p<0.05). A significant difference in the tongue tip displacement was also found between the hold phase and the end phase (F(1,19) = 9.65, p<0.05). As shown in Fig 7, even though no main effect of group was significant, a significant interaction between the phase and the speaker group was observed (F(3,57) = 16.14, p<0.01). Tongue tip displacement relative to the base line in the hold phase was larger in blind than in sighted speakers. Furthermore, following the removal of the acoustic manipulation, sighted speakers moved their tongue tips closer to the baseline position while blind speakers continued to compensate by moving it even further backward.

Finally, results of the tongue dorsum displacement (Fig 8) demonstrated a significant difference between the baseline phase and the hold phase (F(1,19) = 18.19, p<0.01). Even though no main effect of group was found, a significant interaction between the phase and the speaker group was observed (F(3,57) = 14.57, *p*<0.01). As shown in Fig 8, the tongue dorsum displacement was larger for the blind speakers than for the sighted speakers in the hold phase.

To reflect the relation between perceptual and production speech mechanisms, we sought to establish a link between the perceptual abilities of the participants and their degree of compensation to the acoustic manipulation. To do so, we conducted Pearson correlations linking their perceptual scores and their degree of compensation of F2 during the hold phase. No relationship was found between the 50% category boundary, the slope of the labelling function, and the degree of F2 compensation (r (20) = 0.246, p> 0.05).

## Discussion

The goal of this study was to examine the acoustic and articulatory compensatory behaviors in response to real-time manipulations of acoustic feedback among sighted and blind adult speakers. By analyzing the impact of blindness on the acoustic and articulatory behaviors in response to acoustic manipulations, this research assesses the contribution of vision to the speech perception and production mechanisms. To our knowledge, this was the first study to investigate this, and the results supported the first two of our three hypotheses.

Our first hypothesis stated that speakers would alter F2 in the opposite direction of the change they perceive, and that this acoustic compensation would be greater for blind speakers than for sighted speakers (cf. [13]). This hypothesis was confirmed. Indeed, the acoustic adaptation patterns observed in both speaker groups was similar to those reported in the literature [31,33,36,46]. In response to the increase of F2, all participants compensated their vowel productions by reducing the values of F2 of the produced vowels. Moreover, the degree of compensation of F2 varied according to each of the experimental phases. Although some studies have shown that formant manipulation can influence the values of adjacent formants [36,49], we only observed a minor compensation for F1. This result is in line with results found in Purcell and Munhall [33], Villacorta et al. [34] and MacDonald et al. [46], who suggest that control of F1 and F2 are independent [46]. However, when interpreted with the significant decrease of F2, this soft increase in F1 could have allowed the speakers to expand the F1-F2 distance of their productions.

For both speaker groups, a certain number of utterances for which the acoustic feedback was shifted were required before a gradual acoustic compensation occurred. This suggests that a sufficient amount of acoustic manipulation is necessary before participants feel the need to adapt their productions. Otherwise, the perceived targets, although somewhat different from the produced one, remain within their accepted region of tolerance. Similar observations were reported in other acoustic manipulation studies [45,47,48,54].

Although both speaker groups began to adapt their vowel production substantially at the same time, the extent of acoustic compensation was different. Namely, in line with our expectations, blind speakers further adapted the F2 values of their vowel productions. Nevertheless, both speaker groups compensated only partially. For sighted speakers, the adaptation represented 8% of the 500 Hz manipulation while blind speakers compensated up to 14%. Interestingly, this substantial difference between the acoustic compensation of both speaker groups could not be explained by group differences in auditory acuity, since no differences were found in the results of the perceptual tasks. However, as mentioned by Purcell and Munhall [33,39], it remains to be proven whether the detection threshold of an acoustic change in passively perceived speech is comparable to the one we can identify during our own productions. Considering that it is not, larger acoustic compensation found in blind speakers could be the consequence of a smaller detection threshold in their own productions. Similar conclusions were drawn in studies assessing the acoustic compensatory response to formant perturbation across language groups [35,36]. In that context, the authors also suggested that the nature of acoustic compensation, associated with cross-language differences, was phonologically mediated and that it was strongly related to the participant’s phonemic representations such as category prototypes, category goodness and characteristics of vowel perceptual space [35]. Thereby, the divergent acoustic compensation patterns found between the blind and the sighted speakers could reflect a different organization of the internal model ruling the acoustic-motor control of speech [39], which could explain the different degrees of sensorimotory tolerance observed between the two groups. Nevertheless, extensive research is still needed in order for this assumption to be fully established.

Finally, following the removal of the acoustic manipulation, both groups quickly produced vowels similar to those issued during the baseline phase. It is interesting to note that, following the withdrawal of the F2 manipulation, blind participants who had stopped adapting their vowel productions started to compensate once again. We intend to address this aftereffect in future work.

On the articulatory level, the results were also in line with our second hypothesis stating that, during compensated trials, blind speakers would produce larger changes in tongue positions than sighted speakers. Although both speaker groups used all of the articulators to counter the acoustic manipulation, blind and sighted speakers seemed to use different articulatory strategies. Specifically, blind participants were more likely to use the apex and the back of their tongue than their sighted peers to generate changes in the acoustic signal they produced. Those results are in line with previous studies addressing articulatory strategies used by congenitally blind speakers in normal or clear speech [10,11,13], where it was established that, since they do not have access to visual cues, blind speakers mostly rely on non-visible articulators to effect acoustic change.

Our third hypothesis, stating that lip positions would be altered to a lesser extent in blind speakers than in sighted speakers, was not confirmed in the present study. Indeed, while no group difference was observed for the lower lip variable, bigger displacements of the upper lip were found in blind speakers. The principle of motor equivalence in speech production described by Hughes and Abbs [59] provides an interesting explanation for this latter observation. Although they state that motor equivalence in speech patterns is mainly present between the lower lip and the jaw, they showed that compensation coordination can be found in the upper lip in contexts where reduced displacement of the lower lip and the jaw are observed. Supporting this assumption, Abbs and Gracco [60] latter proposed that a principle of synergistic behaviour is present between the upper and lower lip, suggesting that if one is highly recruited, the other will show a lower level of activity.

That said, the fact that more upper lip displacements were found for the blind speakers when the acoustic manipulation was at its peak contrasts with previous results reported by Leclerc [5], Trudeau-Fisette et al. [11] and Ménard et al. [9] showing that, to produce intelligible acoustic targets, sighted speakers mostly used their lips while blind speakers took advantage of their tongues. Moreover, Ménard et al. [9] showed that this articulatory dissimilarity between blind and sighted speakers was even more true when targeted vowels were phonologically contrasted in both rounding and place of articulation, as those involved in the current study.

These seemingly contradictory results can however be reconciled if we consider that in the auditory perturbation experiment, the integrity of the speech target is jeopardized. Thus, if the somatosensory goal has primacy over the auditory goal, speakers will tolerate minimal discrepancies between the expected articulatory positions usually associated with that phoneme and the new positions necessary to compensate, but will accept correspondingly large differences in acoustic-auditory values. In contrast, if the auditory goal has primacy over the somatosensory ones, it is likely that small differences between the production of the acoustic baseline and the compensatory response perceived during the manipulated phases will be observed, but that larger differences in articulatory position will be tolerated. Keeping in mind the complexity of the processes involved in compensation to auditory feedback perturbation (generation of articulatory commands through feedforward commands, internal feedback projection, comparison and error corrections, etc.), we can suggest that in the current study blind speakers tolerated larger somatosensory discrepancies and smaller auditory discrepancies compared to their sighted peers. It is likely that vision contributed to narrower somatosensory targets associated with phonemes for sighted speakers. Our results can be interpreted in line with cross-modal integration studies suggesting that the lack of visual input at an early age would affect somatosensory discrimination [61]. Indeed, according to this hypothesis, in the course of sensory development, the more accurate sense would calibrate the others. In the present study, the fact that our speakers were congenitally blind deprived them of an important source of information, which may have been detrimental to the further development of somatosensory perception. Thus, their somatosensory goal could be larger than it is for sighted speakers. Of course, other differences could be involved in this pattern of results. For instance, somatosensory acuity could differ between sighted and blind speakers. Furthermore, recent neuroimaging studies have suggested contradictory views on the way forward models interact. While one view proposes that parallel pathways operate between motor to somatosensory and motor to auditory cortex [22,24] a serial model incorporates forward models from motor to somatosensory then to auditory cortex [23,25]. Additional neuroimaging data are needed to investigate this issue.

## Conclusion

This study showed that blind and sighted speakers responded differently to a real-time manipulation of their auditory feedback. An observation of the weight given to auditory feedback in an acoustic manipulation task showed that blind participants granted more importance to the auditory information than their sighted peers.
